# A sex-specific genome-wide association study of depression phenotypes in UK Biobank

**DOI:** 10.1038/s41380-023-01960-0

**Published:** 2023-02-07

**Authors:** Patrícia Pelufo Silveira, Irina Pokhvisneva, David M. Howard, Michael J. Meaney

**Affiliations:** 1 Ludmer Centre for Neuroinformatics and Mental Health, Department of Psychiatry, Faculty of Medicine & Douglas Research Centre, McGill University, Montreal, QC Canada; 2 Yong Loo Lin School of Medicine, National University of Singapore, Singapore, Singapore; 3 Social, Genetic and Developmental Psychiatry Centre, Institute of Psychiatry, Psychology & Neuroscience, King’s College London, London, UK; 4 Division of Psychiatry, University of Edinburgh, Royal Edinburgh Hospital, Edinburgh, UK; 5 Translational Neuroscience Program, Singapore Institute for Clinical Sciences and Brain – Body Initiative, Agency for Science, Technology and Research (A*STAR), Singapore, Singapore; 6 Brain–Body Initiative, Institute for Cell & Molecular Biology, Agency for Science, Technology and Research (A*STAR), Singapore, Singapore

**Keywords:** Depression, Genetics

## Abstract

There are marked sex differences in the prevalence, phenotypic presentation and treatment response for major depression. While genome-wide association studies (GWAS) adjust for sex differences, to date, no studies seek to identify sex-specific markers and pathways. In this study, we performed a sex-stratified genome-wide association analysis for broad depression with the UK Biobank total participants (*N* = 274,141), including only non-related participants, as well as with males (*N* = 127,867) and females (*N* = 146,274) separately. Bioinformatics analyses were performed to characterize common and sex-specific markers and associated processes/pathways. We identified 11 loci passing genome-level significance (*P* < 5 × 10^−8^) in females and one in males. In both males and females, genetic correlations were significant between the broad depression GWA and other psychopathologies; however, correlations with educational attainment and metabolic features including body fat, waist circumference, waist-to-hip ratio and triglycerides were significant only in females. Gene-based analysis showed 147 genes significantly associated with broad depression in the total sample, 64 in the females and 53 in the males. Gene-based analysis revealed “Regulation of Gene Expression” as a common biological process, but suggested sex-specific molecular mechanisms. Finally, sex-specific polygenic risk scores (PRSs) for broad depression outperformed total and the opposite sex PRSs in the prediction of broad major depressive disorder. These findings provide evidence for sex-dependent genetic pathways for clinical depression as well as for health conditions comorbid with depression.

## Introduction

Major depressive disorder (MDD) is the leading contributor to the global burden of disease and disability worldwide [1–4]. Depression shows marked sex differences with women significantly more affected in terms of prevalence, repeated occurrence, symptomatology and patterns of co-morbidity [5–12]. The neural correlates of MDD differ in males and females at the level of brain structure [13, 14] and potentially cell-type composition [15]. There are strikingly sex-specific transcriptional signatures of depression in corticolimbic brain regions associated with mood disorders [15–18]. These functional disparities are likely reflected in differences in the response to antidepressant treatment [19–25], which makes the understanding of sex-specific risk factors and vulnerabilities a pressing concern. An understanding of sex-specific pathways to psychopathology is also a fruitful approach to identifying novel mechanisms of pathophysiology [26].

Twin studies reveal evidence for moderate overall heritability for depression [27–29] with some evidence for greater heritability in women and for sex-specific genetic pathways to MDD (e.g., [28, 30]). Despite the evidence for sex-specific molecular mechanisms for depression, genome-wide association studies (GWASs) for MDD are still performed aggregating males and females into a single sample using sex as a covariate. The justification is based on the assumption that large sample sizes are essential and that the analyses of GWA datasets are adjusted by sex. There are two concerns with this approach: (1) pronounced sex differences in transcription suggest that aggregating males and females masks signals that are apparent only when considering datasets from males and females independently, and the noise of combining data regardless of sex might offset any advantage of a larger sample size; (2) collapsing male and female MDD data only permits the identification of molecular signals shared across males and females and thus to an incomplete understanding of the genetic risk factors linked to depression. The risk is that of ignoring critical, sex-specific molecular pathways and targets for the development of new therapies. The existing science suggests sex-specific approaches to treatment and is consistent with the broader objectives of precision medicine. However, to date, this approach in the area of major depression advances in the absence of an understanding of sex-specific genetic pathways.

In this study, we directly explored sex-dependency in the genetic architecture of MDD by performing a follow-up analysis of a published GWAS for “broad depression” [31] after stratifying the analysis by sex. Our findings are consistent with previous twin studies revealing sex-specific genetic architecture to clinical depression, and implicate sex-specific molecular pathways, aligned with genome-wide transcriptomic analyses [32].

## Subjects and methods

### Study population

The UK Biobank cohort is a population-based cohort consisting of 502,543 individuals aged 37–73 recruited at 23 centers across the United Kingdom between 2006 and 2010. Participants provided both phenodata and genodata. Genotyping data were available for 487,409 subjects. We excluded participants who withdrew their consent, with inconsistencies in genetic and reported sex, as well as outliers for heterozygosity. Also, we retained only those subjects who identified themselves as “Caucasians”. After applying the above-mentioned criteria, there were 408,577 subjects. We next excluded 132,066 participants with shared relatedness of up to the third degree (kinship coefficients >0.044 calculated using the KING software). We also removed variants with minor allele frequency <0.01, an imputation accuracy Info score <0.1 as well as duplicated and ambiguous SNPs. As a result, there were 276,511 individuals and 7,351,435 variants in the dataset. The broad depression phenotype was defined according to Howard et al. [31]. Briefly, broad depression was defined using self-reported help-seeking behavior for mental health difficulties. Case and control status were determined by the touchscreen response “yes” to either of these two questions: “Have you ever seen a general practitioner (GP) for nerves, anxiety, tension or depression?” (UK Biobank field: 2090) or “Have you ever seen a psychiatrist for nerves, anxiety, tension or depression?” (UK Biobank field 2010). A case was defined if the participant responded yes at either the initial assessment visit, at any repeat assessment visit, or if there was a primary or secondary diagnosis of a depressive mood disorder from linked hospital admission records (UK Biobank fields: 41202 and 41204; ICD codes: F32—Single Episode Depression, F33—Recurrent Depression, F34—Persistent mood disorders, F38—Other mood disorders and F39—Unspecified mood disorders). This definition is likely to include individuals with internalizing disorders other than depression and those with depressive symptoms that would not meet diagnostic criteria for MDD [31].

Using the broad depression definition resulted in 113,769 cases and 208,811 controls (total = 322,580, prevalence = 35.27%). There were 274,141 unrelated subjects with both the broad depression phenotype and genotype data. From the subsample of related subjects (132,066 participants) we selected one participant per each group of related participants (genetic relatedness <0.025) based on the genomic relationship matrix (calculated using Genome-wide Complex Trait Analysis (GCTA 1.93.2)), which resulted in 65,285 subjects with the broad depression phenotype that served as the test sample. This research was conducted using the UK Biobank Resource under Application Number 41975. Informed written consent was obtained from all participants. Approval for the UK Biobank was obtained by the North West Multicentre Research 580 Ethics Committee (REC reference 11/NW/0382; www.ukbiobank.ac.uk/ethics/), the National Information Governance Board for Health and Social Care and the Community Health Index Advisory Group.

### Association analysis

We applied linear regression analysis using BGENIE v1.132 to explore the effect of each SNP on the broad depression phenotype. Before performing the regression analysis, we adjusted the outcome for sex, age, genotyping array and first eight genetic principal components. Linkage Disequilibrium Score regression (LDSR) [33, 34] was used to determine whether there was an elevation of the polygenic signal due to population stratification, by examining the intercept for evidence of significant deviation (±1.96 standard error) from 1. The genomic inflation factor (λGC) was also reported for each sample. Genetic correlations were calculated between the MDD phenotype for each sex and 237 other behavioral and disease-related traits using LD Hub [33]. *P* values were false discovery rate (FDR) adjusted using the Benjamini and Hochberg approach. We also specifically investigated the genetic correlations between our sex-specific GWASes and a CRP GWAS [35] using LD score regression [34]. Statistical analysis was conducted using RStudio [36] and also included GenomicSEM package [37]. Two-tailed hypothesis tests were considered and the data were verified to ensure that the assumptions for logistic regression analysis are met. The significance level for the analyses including PRSs was set at alpha <0.05.

### Gene-based analysis

Gene- and region-based analyses of the significant genes (*P* < 2.6 × 10^–6^) were conducted using MAGMA (Multi-marker Analysis of GenoMic Annotation) available on FUMA_GWAS (Functional Mapping and Annotation of Genome-Wide Association Studies) [38]. For gene-set pathway analysis, we used the results obtained from the gene-based analysis considering SNPs at 10^–5^ as the threshold to conduct a further gene-set pathway analysis to test for gene enrichment using FUMA_GWAS (Functional Mapping and Annotation of Genome-Wide Association Studies), Gene2func, gene-set analysis, GO molecular functions. We also compared male and female enrichment analyses using MetaCore™ (Clarivate Analytics, version 21.4) (https://portal.genego.com). We used “Particular set” sorting method in MetaCore and exported significant common elements (FDR < 0.05) for comparison purposes. Networks were constructed for direct interactions between selected objects.

Expression quantitative loci (eQTL) identification was performed using data from the online GTEx portal (https://www.gtexportal.org/home/) to determine whether variants at 10^–7^ threshold for phenotype were eQTL in male- and female-specific broad MDD GWAS, focusing on the central nervous system datasets (amygdala, anterior cingulate cortex, caudate, cerebellar hemisphere, cerebellum, cortex, frontal cortex, hippocampus, hypothalamus, nucleus accumbens, putamen, spinal cord, substantia nigra). Transcription factor analysis of the genes mapped from SNPs from male- and female-specific broad MDD GWAS at a p-threshold 10^–5^ were performed using MetaCore™.

We utilized a drug-target network-building tool called Drug Targetor (drugtargetor.com) [39] to establish the potential mechanisms by which antidepressants act in male- vs. female-specific MDD. This resource uses Summary-PrediXcan (a statistical tool that assesses the mediating effects of gene information from summary statistics of genetic association studies on phenotypes; S-PrediXcan) from GWAS databases and drug/target interactions to assess phenotype-informed drug-target networks. The GWAS used was DEPR01: major depressive disorder [40]. The analysis was set to the nervous system, the drug class to antidepressants, and the connection type to bioactivity and gene expression. We selected the maximum number of drugs possible (1500) and 50 drug targets. The gene targets from this analysis were further assessed using the “compare gene list” functions in MetaCore® to determine the relation of male- or female-specific MDD-associated genes altered by antidepressant medications.

### Validation of the sex-specific GWAS through polygenic risk scores

We then aimed at comparing the predictive capacity of the sex-specific MDD polygenic risk scores for detecting broad depression. For the sake of this comparison, we aligned the sample size of the male, female and total GWASs to avoid a power bias. Therefore, we selected ten random subsamples of the UK Biobank participants: one with the sample size similar to the males (129k participants) and another with the sample size similar to the females (147k participants) and conducted GWAS analysis on each. In all subsamples we maintained the same proportion of cases/controls and males/females as in the full UK Biobank sample retained for the analysis (274,141 subjects). As in the main analysis, for every selected subsample we applied linear regression (BGENIE) to access effect of each SNP on the adjusted broad depression phenotype. We then used the GWAS results to calculate the polygenic risk scores at different *P* value thresholds using PRSice software [41, 42] for each subject of the test sample (*N* = 65,285 UK Biobank subjects not originally included in the main GWAS). For the comparison of the predictive ability of the different PRSs, we pooled together the results of the logistic regressions (10 for the females and 10 for the male sample). Logistic regression analysis was applied to explore the associations between PRSs and broad MDD outcome, adjusting for sex, age, genotyping array, assessment center and population stratification. We used Akaike information criterion (AIC) to compare models with different PRSs. This method identifies the best-fit model as the one that explains the greatest amount of variation using the fewest possible independent variables. Lower AIC scores associate with better-fitting models. Finally, as there is abundant evidence confirming that depression is associated with higher inflammation [43–45], we investigated the association between the sex-specific PRS and C-reactive protein (CRP) in males and females from our study. Serum CRP levels were measured by immunoturbidimetric high-sensitivity analysis on a Beckman Coulter AU5800 in UKB. CRP level was log-transformed to account for the highly skewed distribution.

## Results

### Broad depression MDD GWAS in the total sample

**Fig. 1:**
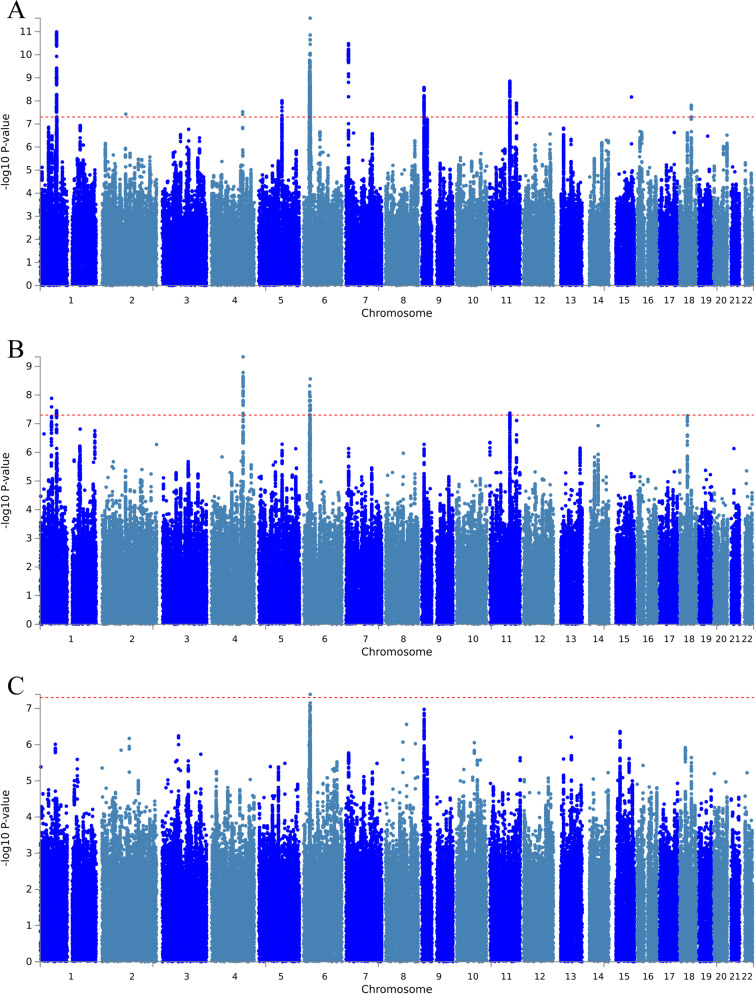
Manhattan plot of all the variants analyzed in UK Biobank for broad depression. **A** total sample (*N* = 274,141), **B** females (*N* = 146,274), and **C** males (*N* = 127,867).

The total sample size (male + female) was *N* = 274,141 including only non-related participants. Broad depression was based on self-reported help-seeking behavior for mental health difficulties from either a general practitioner or psychiatrist (see Supplementary Table S1A for case/control demographics). A total of 18 independent loci showed genome-wide significance associated with broad depression (*P* < 5 × 10^−8^) (Supplementary Data S1 and Fig. 1A). The correlation of the beta coefficients between the Howard et al. [31] broad depression GWAS and our broad depression GWAS was *r* = 0.91 and the correlation between the *P* values was *r* = 0.72. There were 2819 variants with *P* < 10^−6^ for an association with broad depression for the total sample (Fig. 1A and Supplementary Fig. S1). The phenotype examined did not show evidence of inflation of the test statistics due to population stratification, with any inflation due to polygenic signal (see Supplementary Table S2). Genetic correlations from our total broad depression GWAS also replicated the findings from Howard et al. [31] (Table 1 and Supplementary Data S2). There were 35 significant correlations for broad depression with other traits (*P*_FDR_ < 0.05; Supplementary Data S2). Correlations previously described in Howard et al. [31] between UK Biobank depression-related phenotypes and clinically defined MDD as well as schizophrenia [46] (*r*_*g*_ = 0.30) and bipolar disorder [47] (*r*_*g*_ = 0.34).

**Table 1:** Genetic correlations comparison between original Howard et al., 2018 broad MDD GWAS, current study total sample GWAS, female-specific and male-specific broad MDD GWAS.

		Broad MDD GWAS: Howard et al., 2018				Broad MDD GWAS: total sample				Broad MDD GWAS: females				Broad MDD GWAS: males			
Trait	PubMed number	*r*_*g*_	SEM	*P* value	FDR *P* value	*r*_*g*_	SEM	*P* value	FDR *P* value	*r*_*g*_	SEM	*P* value	FDR *P* value	*r*_*g*_	SEM	*P* value	FDR *P* value
Age of first birth	27798627	–0.2981	0.0319	9.07E–21	2.67E–19	–0.2616	0.0339	1.23E–14	2.63E–13	–0.2751	0.0379	4.11E–13	1.07E–11	–0.2238	0.0448	5.73E–07	1.22E–05
Bipolar disorder	21926972	0.3288	0.0366	2.53E–19	6.61E–18	0.3364	0.0381	1.16E–18	3.41E–17	0.2671	0.0466	9.62E–09	1.61E–07	0.3995	0.0559	8.84E–13	2.97E–11
Body fat	26833246	0.1782	0.0402	9.19E–06	1.20E–04	0.1719	0.0403	1.96E–05	2.42E–04	0.2175	0.0485	7.32E–06	8.60E–05	0.0926	0.0469	4.84E–02	3.11E–01
College completion	23722424	–0.2069	0.0384	6.83E–08	1.07E–06	–0.2013	0.0405	6.72E–07	1.05E–05	–0.2556	0.0413	6.11E–10	1.20E–08	–0.1175	0.0613	5.54E–02	3.34E–01
Coronary artery disease	26343387	0.1236	0.0288	1.71E–05	1.98E–04	0.122	0.0304	5.90E–05	6.60E–04	0.1356	0.0375	3.00E–04	2.71E–03	0.1004	0.0407	1.36E–02	1.46E–01
Depressive symptoms	27089181	0.8268	0.0313	1.11E–153	2.61E–151	0.8415	0.0339	9.31E–136	1.09E–133	0.8197	0.0452	1.56E–73	1.83E–71	0.8424	0.0542	2.09E–54	2.46E–52
Ever vs never smoked	20418890	0.3161	0.046	6.56E–12	1.19E–10	0.2945	0.0481	8.82E–10	1.59E–08	0.3286	0.0566	6.39E–09	1.16E–07	0.2395	0.0576	3.16E–05	6.19E–04
Excessive daytime sleepiness	27992416	0.2408	0.043	2.10E–08	3.53E–07	0.2366	0.0458	2.34E–07	3.93E–06	0.2566	0.0556	3.88E–06	5.07E–05	0.2098	0.0597	4.00E–04	6.71E–03
Inflammatory bowel disease (Euro)	26192919	0.1269	0.0342	2.00E–04	1.88E–03	0.132	0.0345	1.00E–04	9.79E–04	0.1275	0.0406	1.70E–03	1.43E–02	0.142	0.0433	1.00E–03	1.57E–02
Insomnia	27992416	0.4379	0.0393	7.63E–29	3.59E–27	0.4303	0.043	1.43E–23	4.80E–22	0.4658	0.0486	9.23E–22	5.42E–20	0.3764	0.0593	2.17E–10	5.67E–09
Insomnia	28604731	0.4013	0.0462	3.82E–18	8.98E–17	0.4085	0.0471	4.42E–18	1.15E–16	0.4463	0.0551	5.45E–16	1.83E–14	0.3507	0.0646	5.63E–08	1.32E–06
Lung cancer	27488534	0.2173	0.0544	6.37E–05	6.81E–04	0.214	0.0555	1.00E–04	9.79E–04	0.2684	0.066	4.80E–05	5.37E–04	0.1261	0.0689	6.73E–02	3.59E–01
Lung cancer (all)	24880342	0.2122	0.0605	5.00E–04	4.35E–03	0.2121	0.0617	6.00E–04	5.42E–03	0.2826	0.0724	9.54E–05	9.34E–04	0.1	0.0779	1.99E–01	5.30E–01
Major depressive disorder	22472876	0.7856	0.0734	9.67E–27	3.79E–25	0.8441	0.0788	8.34E–27	3.92E–25	0.8077	0.086	6.20E–21	2.91E–19	0.8497	0.0984	5.69E–18	2.23E–16
Neuroticism	27089181	0.7266	0.0279	2.25E–149	2.64E–147	0.7414	0.0292	1.30E–142	3.06E–140	0.749	0.035	1.66E–101	3.90E–99	0.7181	0.0419	5.55E–66	1.30E–63
Neuroticism	24828478	0.8204	0.0965	1.92E–17	4.09E–16	0.855	0.0997	9.78E–18	2.30E–16	0.8968	0.1209	1.19E–13	3.50E–12	0.776	0.1089	1.04E–12	3.06E–11
Number of children ever born	27798627	0.1795	0.0409	1.16E–05	1.44E–04	0.1734	0.0438	7.50E–05	8.01E–04	0.1974	0.0494	6.41E–05	6.85E–04	0.1347	0.056	1.61E–02	1.58E–01
PGC cross-disorder analysis	23453885	0.4712	0.0372	1.04E–36	6.14E–35	0.4839	0.0404	4.13E–33	2.43E–31	0.4125	0.0476	4.20E–18	1.64E–16	0.5514	0.0587	5.47E–21	2.57E–19
Schizophrenia	25056061	0.3008	0.0285	5.28E–26	1.77E–24	0.3026	0.0302	1.12E–23	4.39E–22	0.2299	0.0331	3.53E–12	8.30E–11	0.3806	0.0401	2.48E–21	1.46E–19
Squamous cell lung cancer	27488534	0.3099	0.0907	6.00E–04	5.04E–03	0.3001	0.0911	1.00E–03	8.39E–03	0.3799	0.1018	2.00E–04	1.88E–03	0.1613	0.1163	1.65E–01	5.04E–01
Subjective well-being	27089181	–0.6069	0.0406	1.33E–50	1.04E–48	–0.6228	0.0415	6.49E–51	5.08E–49	–0.5937	0.0492	1.77E–33	1.39E–31	–0.6338	0.0583	1.54E–27	1.21E–25
Triglycerides	20686565	0.1183	0.0303	9.17E–05	9.37E–04	0.1318	0.0307	1.73E–05	2.26E–04	0.1529	0.039	8.81E–05	9.00E–04	0.0979	0.0397	1.37E–02	1.46E–01
Waist circumference	25673412	0.1019	0.0277	2.00E–04	1.88E–03	0.1025	0.0297	6.00E–04	5.42E–03	0.1627	0.0346	2.62E–06	3.62E–05	0.0064	0.0385	8.69E–01	9.26E–01
Waist-to-hip ratio	25673412	0.1211	0.0282	1.77E–05	1.98E–04	0.1183	0.0284	3.12E–05	3.67E–04	0.1531	0.0337	5.46E–06	6.75E–05	0.0603	0.0373	1.06E–01	4.46E–01
Years of schooling (proxy cognitive performance)	25201988	–0.1836	0.037	7.15E–07	1.05E–05	–0.1776	0.0384	3.72E–06	5.14E–05	–0.2081	0.0411	4.17E–07	6.53E–06	–0.1227	0.0544	2.41E–02	2.18E–01
Years of schooling 2013	23722424	–0.1768	0.0373	2.16E–06	2.99E–05	–0.1762	0.0379	3.36E–06	4.94E–05	–0.2067	0.0414	5.81E–07	8.53E–06	–0.1192	0.0539	2.71E–02	2.20E–01
Years of schooling 2016	27225129	–0.1703	0.0223	2.08E–14	4.08E–13	–0.1613	0.0229	1.95E–12	3.82E–11	–0.1901	0.0284	2.09E–11	4.47E–10	–0.1139	0.0305	2.00E–04	3.62E–03

### Sex-specific broad depression MDD GWAS

We then stratified our total sample by sex to perform sex-specific GWASs separately for 127,867 non-related male and 146,274 non-related female participants (see Supplementary Table S1B, C for case/control demographics). Results for the associated variants with broad depression phenotype in males and females are provided in Supplementary Data S1, Fig. 1B and S2 for females and 1C and S3 for males. Broad depression in females and males did not show evidence of inflation of the test statistics due to population stratification, with any inflation due to polygenic signal (Supplementary Table S2). Eleven loci passed genome-level significance (*P* < 5 × 10^−8^) in females and one in males GWAS. Interestingly, among these loci was an SNP (rs10501696) that mapped to *GRM5* reported in Howard et al. [31], significant only in females.

### Sex-dependent genetic correlations

Depression is comorbid with a range of other diseases including conditions considered to be not primarily of brain origin, especially cardio-metabolic conditions. Our findings (Table 1) demonstrated 27 and 15 significant (*P*_FDR_ < 0.05) correlations with other traits in females and males, respectively. Genetic correlations between the UK Biobank depression-related phenotypes and clinically defined MDD, schizophrenia [46], bipolar disorder [47], neuroticism [48], subjective well-being [48], PGC cross-disorder [49], insomnia [50, 51] and smoking [52] were significant for both females and males.

Two phenotypic categories showed striking, sex-dependent correlations, each with greater evidence for associations amongst females (Table 1). While analyses with both male and female participants showed associations with measures of academic achievement, the evidence for significant genetic correlations was stronger and more pervasive in females. Significant genetic (*P*_FDR_ < 0.05) correlations only in females included multiple analyses of years of schooling and college completion (*r*_*g*_ = –0.21 and –0.26, respectively) [53]. The most striking sex difference were those between broad MDD and GWASs for metabolic features, including body fat [54], waist circumference [55], waist-to-hip ratio [55] and triglycerides [56], significant at *P*_FDR_ < 0.05 only in females.

The genetic correlation between male and female broad MDD GWAS was *r*_*g*_ = 0.91, *P* = 7.29e–59. We also performed genetic correlations between our sex-specific broad depression MDD GWAS and previously published sex-specific GWAS for ADHD [57] and for fasting insulin [58]. Male-specific broad MDD GWAS correlation with male-specific ADHD GWAS (*r*_*g*_ = 0.26, *P* = 3.68e–05) was significant; however, the correlation with male-specific fasting insulin GWAS (*r*_*g*_ = –0.0013, *P* = 0.99) was not significant. Female-specific broad MDD GWAS significantly correlated with female-specific ADHD GWAS (*r*_*g*_ = 0.51, *P* = 1.21e–09), but not with female-specific fasting insulin GWAS (*r*_*g*_ = 0.13, *P* = 0.17).

### Sex-specific broad MDD GWAS: gene-based enrichment analysis

SNPs with *P* < 10^–5^ were selected to compare female and male GWAs. A total of 147 genes were significantly associated with broad depression in the total sample, 64 in females and 53 in males (Supplementary Figs. S4–S6 and Supplementary Data S3A–C). In addition to *GRM5*, *ELAVL4*, implicated in depression and in epigenome-wide association studies of suicide [59], was significant in females, but not males. In contrast, 29 genes were significant only in males. Notably, *TCF4*, *NKAPL*, *ZKSCAN3*, *ZSCAN16*, *ZSCAN31* have been associated with depression in previous GWASs [40, 60–63]. Twenty-four common genes were found when comparing the list of total, males and females broad MDD GWAS, which were enriched for biological processes related to sensory perception and G protein-coupled receptor signaling pathways.

There were several Pathway Maps in common between genes derived from the male- and female-specific broad MDD GWASs, the most significant of which was glutathione metabolism (*P*_FDR_ < 0.025) (Supplementary Data S4A). Glutathione is involved in antioxidant defense and regulation of gene expression, cell proliferation and apoptosis, signal transduction, and immune response [64]. Several common GO processes were found between males and females, the most significant of which were negative regulation of viral life cycle, regulation of viral release from host cell, regulation of transcription DNA-templated, regulation of nucleic acid-templated transcription, regulation of RNA biosynthetic process (all *P*_FDR_ < 0.002) (Supplementary Data S4B). Common genes between males and females were enriched for diseases related to neurocognitive and neurodegenerative conditions (Supplementary Data S4C).

Enrichment analysis for the genes uniquely identified in male- or female-specific broad MDD GWASs is provided in Supplementary Data S5A–E. Male-specific broad MDD GWAS genes were enriched for several Pathway Maps, GO processes and process networks (Supplementary Data S5A, B, D) with epigenetic regulation of gene expression as the recurrently enriched pathway. Female-specific broad MDD GWAS genes did not show significant enrichment for Pathway Maps but, as in males, were enriched for regulation of gene expression (*P*_FDR_ < 0.01, Supplementary Data S5C).

**Fig. 2:**
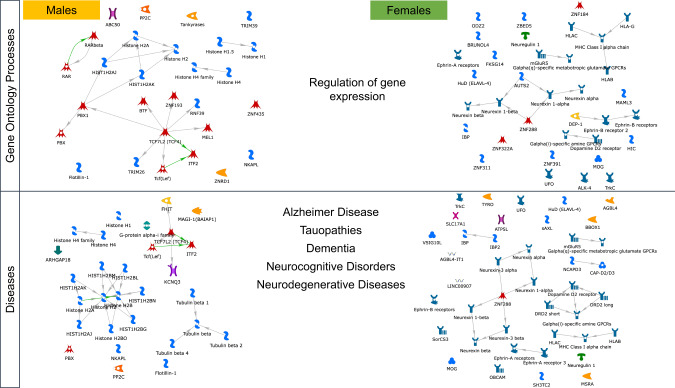
Enrichment analysis of genes associated with male-specific or female-specific broad MDD GWAS. Processes and diseases are commonly enriched in both sexes, but due to unique, sex-specific mechanisms.

It is noteworthy that “regulation of gene expression” was the most significant common GO process associated with genes from both male- and female-specific broad MDD GWAS. However, this finding was due to sex-specific gene networks (Fig. 2). In males, “regulation of gene expression” was mapped to genes including *TCF4* as well as an impressive number of genes coding for histone protein variants. *TCF4* is a known regulator of epigenetic states including DNA methylation [65]. In females, “regulation of gene expression” was associated with a number of neurexin-related genes, *DRD2* and *GRM5* genes.

Several psychopathology and neurodegenerative disease terms were associated with genes from both male- and female-specific broad MDD GWAS (Fig. 2), but this finding was due to sex-specific gene networks. In males, brain pathology was associated with genes involved in epigenetic processes and regulation of neurotransmitter release. In females, brain pathology was related to *DRD2* signaling, and an important number of genes related to adaptive immunity. Taken together, the findings depicted in Fig. 2 suggest that MDD-related alterations in gene expression regulation and brain pathology in males and females occur via unique, sex-specific mechanisms.

### Sex-specific broad MDD GWAS: eQTLs, transcription factors and drug targets

We sought eQTLs among the SNPs identified in male- and female-specific broad MDD GWAS (top 125 significant SNPs, *P* < 10^–7^ in females and *P* < 9.16 × 10^–7^ in males). There was a striking sex difference with almost twice the percentage of SNP/eQTLs in males [18% compared with females 10% (*χ*^2^ = 945.9, *P* < 0.00001)] considering all brain regions together.

The cerebellum showed a high number of eQTLs in both male- and female-specific GWASs (Supplementary Fig. S7). There was a highly sex-specific distribution of identified eQTLs across brain regions. eQTLs from the female-specific GWAs in the caudate-basal ganglia (*χ*^2^ = 38.8; *P* < 0.00001), putamen-basal ganglia (*χ*^2^ = 31.6; *P* < 0.00001), and hippocampus (*χ*^2^ = 19.7; *P* < 0.00001) significantly exceeded that derived from the male-specific GWAs. The most notable finding was that of increased eQTLs in the basal ganglia of females, including the ventral striatum/nucleus accumbens, which are prominent dopamine target regions.

A conspicuous pattern apparent in both males and females was the enrichment for transcription factors linked to immune responses and NFK-ß signaling, including FOXP3, RBPJ kappa, RUNX1 and TAL1. BMAL1, a critical regulator of circadian rhythms [66], was highly enriched amongst the genes from both the male- and female-specific GWASs (Supplementary Data S6A, B). This finding is interesting considering the highly significant genetic correlation between both the male- and female-specific GWASs for broad MDD and that for insomnia (see Table 1).

Transcription factors uniquely associated with genes identified in the female broad MDD GWAS were linked to oxidative stress, apoptosis and type II diabetes (e.g., HNF3-beta, MafA, p63, RelA, VDR), which aligns with the genetic correlation of the female-specific GWAS with cardio-metabolic conditions (see Table 1). Transcription factors linked specifically with genes identified in the male-specific broad MDD GWAS were related to tissue and neuron differentiation as well as epigenetic processes (e.g., E2F1, Esrrb, NRSF, ZFX, ZNF423), which is consistent with the results of the gene enrichment analyses (see Fig. 2) that underscore the potential role for chromatin remodeling factors in males.

The central biological processes associated with broad MDD and targeted by antidepressants were strongly associated with: (1) epigenetic processes such as chromatin assembly, cell cycle regulation as well as inflammation (mainly through IL-7), in males; (2) neuronal migration, regulation of neurotrophic factors and synaptic plasticity, and dopamine neurotransmission, in females (Supplementary Data S7A, B). The implication of the dopamine neurotransmission is consistent with the pathways identified in females in the gene enrichment analyses (Fig. 2), likewise, the finding of chromatin assembly in males, a process intimately linked to histone proteins. These findings suggest that the biological processes targeted by antidepressants and involved in genetic architecture of MDD differ in males and females, implying sex-dependent therapeutic pathways.

### Validation of the sex-specific broad MDD GWAS using polygenic risk scores

**Fig. 3:**
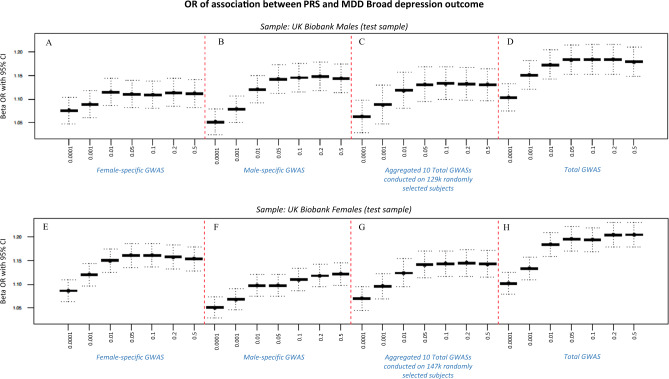
Sex-specific polygenic risk scores (PRS). Comparison between odds ratios (ORs) of associations between the PRS for broad MDD in a test sample of males (**A**–**D**) and females (**E**–**H**) using the female-specific broad MDD GWAS (**A**, **E**), male-specific broad MDD GWAS (**B**, **F**), or a mixed sample MDD GWAS of similar size (147k or 129k, respectively, **C**, **G**). The ORs of associations using the total mixed sample MDD GWAS are shown in panel **D** for male participants and panel **H** for female participants.

We calculated PRSs using our total, male- and female-specific GWASs in a UK Biobank test sample of 65,285 non-related individuals (Supplementary Table S3). In males, the male-specific PRS showed better predictive value for the broad MDD outcome (Fig. 3B) than did the total PRS derived from a similar-sized GWAS (Fig. 3C) or the female-specific PRS (Fig. 3A) (AIC_m_ = 33,962.86, AIC_f_ = 34,001.21, AIC_t_ = 33,977.70). Similarly, the PRS derived from the female-specific GWAS showed better predictive value for the broad depression outcome among females (Fig. 3E) than did the total PRS from a GWAS of comparable size (Fig. 3G) and male-specific PRS (Fig. 3F) (AIC_f_ = 48,987.21, AIC_m_ = 49,100.48, AIC_t_ = 49,016.82). AICs were very similar between our PRSs and polygenic scores calculated with an alternative (PRS-cs) method [67] (male-specific GWAS in the male test sample, AIC PRS-cs = 33,934; female-specific GWAS in the female test sample, AIC PRS-cs = 48,875). The well-established effect of GWAS sample size to best predict the outcomes was observed, as the combined, mixed GWAS sample had a similar predictive capacity to the sex-specific male or female-specific GWAS (Fig. 3D, H). Although a larger GWAS does indeed have a similar predictive capacity than the sex-specific GWAS, it does not have the ability to disentangle sex-specific mechanisms and drug targets.

Finally, in agreement with our genetic correlation analysis that identified sex-specific correlations between female broad MDD GWAS and GWASs for several metabolic features, we observed statistically significant relationships between the female-specific PRS at different thresholds and log CRP levels in females, but not in males (Supplementary Table S4). Interestingly, the sex-specific association identified at the phenotype level was confirmed at the genetic correlation level, as our female-specific broad MDD GWAS correlated with CRP GWAS using LD score regression analysis (*r*_*g*_ = 0.11, *P* = 0.005). The same correlation was not significant in males (*r*_*g*_ = 0.05, *P* = 0.21).

## Discussion

Sex differences in the prevalence and severity of depression are well established. Nevertheless, research to elucidate the genetic architecture of depression has not been designed to examine sex-specific pathways [68]. The current study addresses this gap. To our knowledge, our results are the first extensive molecular analysis of sex-specific genetic pathways for MDD. Our findings reveal evidence for sex differences in the genetic pathways to MDD and are consistent with previous, genetically informed epidemiological studies [27, 28] suggesting that the influences of genetic variants on the risk for depression are sex-specific. We showed that a PRS derived from a female-specific, genome-wide analysis outperformed a version derived from a male-specific analysis in the prediction of broad MDD in females; the reverse was true for the prediction of broad MDD in males (Fig. 3). While the gene enrichment analysis identified “regulation of gene expression” as a common biological process linking genetic variants to broad MDD, the underlying gene pathways were highly sex-dependent (Fig. 2). Regulation of gene expression in males was associated with epigenetic mechanisms, notably variants in genes coding for the core histone proteins. In contrast, regulation of gene expression in females associated with neurexin as well as the DRD2 and mGluR5 receptors.

The implication of the DRD2 receptor in the “regulation of gene expression” pathway is consistent with previous reports. In their MDD GWAS Howard et al. [31] explored drug–gene interactions and identified the DRD2 receptor as the primary target. Levey et al. [69] mapped genes from their GWAS meta-analysis of depression to expression QTL data in GTEx revealing a transcriptome-wide association with a predicted decrease in nucleus accumbens DRD_2_ expression. Our findings suggest that these results might be largely driven by data from women. Note the inclusion of the DRD2 receptor in the female-specific “Regulation of Gene Expression Pathway” (Fig. 2). Our gene-based eQTL analysis of the female-specific GWAS revealed enrichment for eQTLs in the caudate, which includes the dorsal and ventral striatum/nucleus accumbens, brain regions rich in DRD2 receptors.

Considerable evidence from both pre-clinical and clinical studies show the importance of dopaminergic projections from the ventral tegmental area (VTA) to nucleus accumbens for depression [70]. Anhedonia is a core feature of depression and the mesolimbic dopamine pathway mediates activation of the reward system [71]. Deep brain stimulation affecting the nucleus accumbens has sustained efficacy in treatment-resistant depression [72]. The mesolimbic dopamine pathway is critical for chronic stress-induced depressive-like behaviors in rodents [73–75]. Buproprion, a mixed norepinephrine/dopamine-reuptake inhibitor, is an accepted treatment for depression with efficacy comparable to that of SSRIs [76, 77]. Pre-clinical studies reveal greater DRD2-mediated reward-enhancing effects of buproprion in females than males [78]. Rodent studies consistently show sex differences in dopaminergic systems in relation to reward processing and activation of behavioral responses to stress [79]. Human PET imaging studies using [11C]raclopride, a DRD2/3-specific ligand, show in vivo evidence for stress-induced DRD2 signaling in the ventral striatum [80] and decreased ventral striatal D2 synaptic activity in depressed patients [81]. Human neuroimaging studies reveal greater stress-induced activation of the ventral striatum in women compared to men [82, 83].

Eleven loci passed genome-level significance (*P* < 5 × 10^−8^) in females including an SNP (rs10501696) mapped to *GRM5* also reported in Howard et al. [31]. Wray et al. [40] reported on a meta-analysis of seven independent cohorts and identified a significant association of *GRM5* with MDD. *GRM5* gene, which encodes the mGluR5 metabotropic glutamate receptor, was also featured in the female-specific “Regulation of Gene Expression Pathway” (Fig. 2). These sex-specific *GRM5* findings are consistent with Gray et al. [84] report of extensive sex-dependent differences in glutamate receptor gene expression in post-mortem samples from MDD and control subjects, and higher expression levels of *GRIN1, GRIN2A-D, GRIA2-4, GRIK1-2, GRM1, GRM4, GRM5* and *GRM7* in female MDD patients; amongst males only *GRM5* expression differed and in the opposite direction from females.

The G protein-coupled mGluR5 receptor is located both pre- and post-synaptically, regulating synaptic plasticity [85]. mGluR5-/- mice show increased stress-induced depressive-like behaviors [86]. Reductions in mGluR5 protein levels are apparent in multiple rodent models of depression [87, 88], in contrast to the increases in mGluR5 levels observed following antidepressant treatments [89]. In vivo PET imaging with [11C]ABP688 reveals lower mGluR5 receptor density in MDD in several regions [90], which seems to be associated with a response to antidepressant treatment [91].

Our gene level analysis identified “regulation of gene expression” as the primary biological process for both males and females, revealing the involvement of neurexin signaling in females (Fig. 2), which is critical for the formation of neural circuits and implicated in a range of neuropsychiatric disorders [92, 93]. To our knowledge, previous GWASs have not provided evidence for an association between polymorphisms in neurexin genes and MDD. However, alternative gene sequencing platforms do suggest a potential role for neurexin in MDD. Rucker et al. [94] found significant enrichment of genomic and exonic deletion CNVs in cases of recurrent depression. The analysis showed overlap with CNVs previously associated with schizophrenia, including neurexin 1. This same exonic *NRXN1* deletion CNV was also associated with a poor treatment response to antidepressants [95]. An intronic SNP in neurexin 3 was significantly associated with symptom improvement following citalopram/escitalopram treatment [96]. Transcriptomic analyses with post-mortem human brain samples reveal significant sex differences in both the expression and splicing of neurexin genes [97, 98]. Studies with model systems suggest a prominent role for neurexin in establishing and maintaining sexually dimorphic neuronal circuits [99].

The “Regulation of Gene Expression” process in males was associated with histone modifications. An analysis from the Psychiatric Genomics Consortium [100] examined common pathways across GWASs for schizophrenia, major depression and bipolar disorder, identifying histone methylation as the strongest emerging common process. Pre-clinical models of depression underscore the importance of histone methylation [101, 102], with emerging evidence from human post-mortem analyses [103]. Our analysis identified genes coding for actual histone protein variants rather than enzymes associated with methylation. Histone protein variants emerge from a histone gene cluster as key components of the transcriptional machinery [104]. Histone variants affect nucleosome dynamics associated with activity-dependent transcription in the brain [105, 106] and differ in the capacity for post-translational modification. Hodes et al. [107] summarized the existing evidence for sex-dependent effects of epigenetic mechanisms in rodent models of depression. The extensive sex-dependent transcriptomic profiles of depression together with the sex differences in eQTLs described here point to an important area for future analyses.

While we emphasize the sex-dependent features of our dataset, there were important points of convergence between male and female analyses. Our transcription factor enrichment analysis revealed common transcriptional signals, most notably factors involved in NFK-ß-signaling such as FOXP3, TAL1 and RUNX1, consistent with the proposed association between inflammation and MDD [108]. Another common factor was BMAL1, a regulator of circadian rhythms and sleep. Genetic correlation between the broad MDD GWAS and insomnia was significant in both sexes (Table 1). Notably absent in the transcription factor enrichment analysis were sex-steroid receptors, which serve as ligand-gated regulators of gene expression.

Genetic correlations for male- and female-specific GWASs revealed significant associations with previous GWASs for a range of psychiatric disorders (see Table 1). Nevertheless, a striking sex difference in the genetic correlations was those between broad MDD and GWASs for metabolic features, including body fat, waist circumference, waist-to-hip ratio and triglycerides, all of which associate with an increased risk for cardio-metabolic disease and were highly correlated in women, not men (Table 1). Interestingly, we also observed sex-specific associations between the female broad MDD PRS and serum CRP in women, but not in men, a finding that was confirmed a significant genetic correlation between the female-specific broad MDD GWAS and the CRP GWAS. The genetic correlation between the male-specific broad MDD GWAS and CRP GWAS was not significant. This finding partially replicates the association previously described in [43], in which a PRS for major depressive disorder was positively associated with CRP level. Our study suggests that the low-grade inflammation associated with depression is linked to the genetic background related to MDD in females, but not in males. Co-morbidity between MDD and cardio-metabolic diseases is well established [109] being significantly more prevalent in women [110–113] and see [114] for a review]. Marcus et al. [115] reported that women with MDD were more likely to endorse changes in appetite and weight gain than were men in the STAR*D (www.star-d.org) trial. Our findings suggest that sex-specific genetic pathways may explain, in part, this increased co-morbidity in women. The sex-specific MDD pathway analyses highlighted dopamine signaling in females and analyses of the sex-specific GWASs showed greater eQTLs in the basal ganglia in women, including the caudate and nucleus accumbens. Dopamine signaling through DRD2 receptors in these regions regulates appetite and feeding behavior [116].

Our study was an exploratory investigation into potential sex-dependent genetic pathways to depression. Our genome-wide analyses were not sufficiently powered to provide definitive identification of sex-dependent loci associated with MDD, requiring extension with sex-based stratification of larger sample sizes and diverse populations. Nevertheless, we note that despite the comparatively smaller sample size, the female-specific GWAS did yield 11 loci that passed genome-wide statistical significance. A comparably powered analysis failed to yield significant loci in the male-specific GWAS. Sex-stratified analyses with larger sample sizes are required to examine whether this reflects a greater genetic contribution to MDD in women.

The reliance on a self-reported, “broad MDD” designation is a limitation of this exploratory study. However, Howard et al. [31] showed a highly significant genetic correlation (*r*_*g*_ = 0.86) between broad depression and clinically diagnosed MDD [also see [117]]. The analyses are also limited by the focus on British Caucasians, with subjects generally exceeding the health and wealth of the overall British population.

In summary, our exploratory study suggests that the genetic background linked to human major depression includes sex-specific variants. There are both common and unique biological mechanisms mapped from male-specific and female-specific broad MDD GWAS. Common processes like regulation of gene expression and diseases like brain pathology emerged from sex-specific gene networks. Our findings may contribute to the development of tailored therapeutic options. The consideration of sex-specific molecular alterations related to major depression during disease management can lead to a more effective response to antidepressants. Significantly larger samples across more diverse populations will be required to meet this objective. Our results are intended to add to the rationale for studies of sex-specific mechanisms.

### Supplementary information

Supplementary tables

Supplementary figures

Supplementary Data 1

Supplementary Data 2

Supplementary Data 3

Supplementary Data 4

Supplementary Data 5

Supplementary Data 6

Supplementary Data 7

## Data Availability

The raw genetic and phenotypic data that support the findings of this study are available from UK Biobank but restrictions apply to the availability of these data, which were used under license for the current study, and so are not publicly available. Data are, however, available from the authors upon reasonable request and with permission of UK Biobank (http://www.ukbiobank.ac.uk/).

## Code availability

The code used for analysis is available upon request.

## References

[1] Vos T, Barber RM, Bell B, Bertozzi-Villa A, Biryukov S, Bolliger I (2015). Global, regional, and national incidence, prevalence, and years lived with disability for 301 acute and chronic diseases and injuries in 188 countries, 1990-2013: a systematic analysis for the Global Burden of Disease Study 2013. Lancet.

[2] James SL, Abate D, Abate KH, Abay SM, Abbafati C, Abbasi N (2018). Global, regional, and national incidence, prevalence, and years lived with disability for 354 diseases and injuries for 195 countries and territories, 1990-2017: a systematic analysis for the Global Burden of Disease Study 2017. Lancet.

[3] Smith K (2014). Mental health: a world of depression. Nature.

[4] Rehm J, Shield KD (2019). Global burden of disease and the impact of mental and addictive disorders. Curr Psychiatry Rep.

[5] Weissman MM, Klerman GL (1977). Sex differences and the epidemiology of depression. Arch Gen Psychiatry.

[6] Frank E, Carpenter LL, Kupfer DJ (1988). Sex differences in recurrent depression: are there any that are significant?. Am J Psychiatry.

[7] Young MA, Fogg LF, Scheftner WA, Keller MB, Fawcett JA (1990). Sex differences in the lifetime prevalence of depression: does varying the diagnostic criteria reduce the female/male ratio?. J Affect Disord.

[8] Kessler RC, McGonagle KA, Swartz M, Blazer DG, Nelson CB (1993). Sex and depression in the National Comorbidity Survey. I: lifetime prevalence, chronicity and recurrence. J Affect Disord.

[9] Kessler RC, Berglund P, Demler O, Jin R, Merikangas KR, Walters EE (2005). Lifetime prevalence and age-of-onset distributions of DSM-IV disorders in the National Comorbidity Survey Replication. Arch Gen Psychiatry.

[10] Gater R, Tansella M, Korten A, Tiemens BG, Mavreas VG, Olatawura MO (1998). Sex differences in the prevalence and detection of depressive and anxiety disorders in general health care settings: report from the World Health Organization Collaborative Study on Psychological Problems in General Health Care. Arch Gen Psychiatry.

[11] Marcus SM, Young EA, Kerber KB, Kornstein S, Farabaugh AH, Mitchell J (2005). Gender differences in depression: findings from the STAR*D study. J Affect Disord.

[12] MacKenzie CS, Reynolds K, Cairney J, Streiner DL, Sareen J (2012). Disorder-specific mental health service use for mood and anxiety disorders: associations with age, sex, and psychiatric comorbidity. Depress Anxiety.

[13] Hastings RS, Parsey RV, Oquendo MA, Arango V, Mann JJ (2004). Volumetric analysis of the prefrontal cortex, amygdala, and hippocampus in major depression. Neuropsychopharmacology.

[14] Kong L, Chen K, Womer F, Jiang W, Luo X, Driesen N (2013). Sex differences of gray matter morphology in cortico-limbic-striatal neural system in major depressive disorder. J Psychiatr Res.

[15] Seney ML, Huo Z, Cahill K, French L, Puralewski R, Zhang J (2018). Opposite molecular signatures of depression in men and women. Biol Psychiatry.

[16] Labonté B, Engmann O, Purushothaman I, Menard C, Wang J, Tan C (2017). Sex-specific transcriptional signatures in human depression. Nat Med.

[17] Issler O, van der Zee YY, Ramakrishnan A, Wang J, Tan C, Loh YHE (2020). Sex-specific role for the long non-coding RNA LINC00473 in depression. Neuron.

[18] Seney ML, Glausier J, Sibille E (2022). Large-scale transcriptomics studies provide insight into sex differences in depression. Biol Psychiatry.

[19] Kornstein SG, Schatzberg AF, Thase ME, Yonkers KA, McCullough JP, Keitner GI (2000). Gender differences in treatment response to sertraline versus imipramine in chronic depression. Am J Psychiatry.

[20] Martényi F, Dossenbach M, Mraz K, Metcalfe S (2001). Gender differences in the efficacy of fluoxetine and maprotiline in depressed patients: a double-blind trial of antidepressants with serotonergic or norepinephrinergic reuptake inhibition profile. Eur Neuropsychopharmacol.

[21] Khan A, Brodhead AE, Schwartz KA, Kolts RL, Brown WA (2005). Sex differences in antidepressant response in recent antidepressant clinical trials. J Clin Psychopharmacol.

[22] Berlanga C, Flores-Ramos M (2006). Different gender response to serotonergic and noradrenergic antidepressants. A comparative study of the efficacy of citalopram and reboxetine. J Affect Disord.

[23] Young EA, Kornstein SG, Marcus SM, Harvey AT, Warden D, Wisniewski SR (2009). Sex differences in response to citalopram: a STAR*D report. J Psychiatr Res.

[24] Sramek JJ, Murphy MF, Cutler NR (2016). Sex differences in the psychopharmacological treatment of depression. Dialogues Clin Neurosci.

[25] LeGates TA, Kvarta MD, Thompson SM (2019). Sex differences in antidepressant efficacy. Neuropsychopharmacology.

[26] Rutter M, Caspi A, Moffitt TE (2003). Using sex differences in psychopathology to study causal mechanisms: unifying issues and research strategies. J Child Psychol Psychiatry.

[27] Kendler KS, Gardner CO, Neale MC, Prescott CA (2001). Genetic risk factors for major depression in men and women: similar or different heritabilities and same or partly distinct genes?. Psychol Med.

[28] Kendler KS, Gatz M, Gardner CO, Pedersen NL (2006). A Swedish national twin study of lifetime major depression. Am J Psychiatry.

[29] Flint J, Kendler KS (2014). The genetics of major depression. Neuron.

[30] Kendler KS, Prescott CA, Myers J, Neale MC (2003). The structure of genetic and environmental risk factors for common psychiatric and substance use disorders in men and women. Arch Gen Psychiatry.

[31] Howard DM, Adams MJ, Shirali M, Clarke TK, Marioni RE, Davies G (2018). Genome-wide association study of depression phenotypes in UK Biobank identifies variants in excitatory synaptic pathways. Nat Commun.

[32] Wu W, Howard D, Sibille E, French L (2021). Differential and spatial expression meta-analysis of genes identified in genome-wide association studies of depression. Transl Psychiatry.

[33] Zheng J, Erzurumluoglu AM, Elsworth BL, Kemp JP, Howe L, Haycock PC (2017). LD Hub: a centralized database and web interface to perform LD score regression that maximizes the potential of summary level GWAS data for SNP heritability and genetic correlation analysis. Bioinformatics.

[34] Bulik-Sullivan B, Loh PR, Finucane HK, Ripke S, Yang J, Patterson N (2015). LD Score regression distinguishes confounding from polygenicity in genome-wide association studies. Nat Genet.

[35] Ligthart S, Vaez A, Võsa U, Stathopoulou MG, de Vries PS, Prins BP (2018). Genome analyses of >200,000 individuals identify 58 loci for chronic inflammation and highlight pathways that link inflammation and complex disorders. Am J Hum Genet.

[36] R Core Team. A language and environment for statistical computing. R Foundation for Statistical Computing, Vienna, Austria. 2021. https://www.R-project.org/.

[37] Grotzinger A, van der Zee M, Rhemtulla M, Ip H, Nivard M, Tucker-Drob E. GenomicSEM: structural equation modeling based on GWAS summary statistics. R Packag Version 0.0.5. 2022.

[38] Watanabe K, Taskesen E, Van Bochoven A, Posthuma D (2017). Functional mapping and annotation of genetic associations with FUMA. Nat Commun.

[39] Gaspar HA, Hübel C, Breen G (2019). Drug Targetor: a web interface to investigate the human druggome for over 500 phenotypes. Bioinformatics.

[40] Wray NR, Ripke S, Mattheisen M, Trzaskowski M, Byrne EM, Abdellaoui A (2018). Genome-wide association analyses identify 44 risk variants and refine the genetic architecture of major depression. Nat Genet.

[41] Choi SW, O’Reilly PF (2019). PRSice-2: Polygenic Risk Score software for biobank-scale data. Gigascience.

[42] Chen LM, Yao N, Garg E, Zhu Y, Nguyen TTT, Pokhvisneva I (2018). PRS-on-Spark (PRSoS): a novel, efficient and flexible approach for generating polygenic risk scores. BMC Bioinforma.

[43] Pitharouli MC, Hagenaars SP, Glanville KP, Coleman JRI, Hotopf M, Lewis CM (2021). Elevated C-reactive protein in patients with depression, independent of genetic, health, and psychosocial factors: results from the UK Biobank. Am J Psychiatry.

[44] Osimo EF, Baxter LJ, Lewis G, Jones PB, Khandaker GM (2019). Prevalence of low-grade inflammation in depression: a systematic review and meta-analysis of CRP levels. Psychol Med.

[45] Osimo EF, Pillinger T, Rodriguez IM, Khandaker GM, Pariante CM, Howes OD (2020). Inflammatory markers in depression: a meta-analysis of mean differences and variability in 5166 patients and 5083 controls. Brain Behav Immun.

[46] Ripke S, Neale BM, Corvin A, Walters JTR, Farh KH, Holmans PA (2014). Biological insights from 108 schizophrenia-associated genetic loci. Nature.

[47] Sklar P, Ripke S, Scott LJ, Andreassen OA, Cichon S, Craddock N (2011). Large-scale genome-wide association analysis of bipolar disorder identifies a new susceptibility locus near ODZ4. Nat Genet.

[48] Okbay A, Baselmans BML, De Neve JE, Turley P, Nivard MG, Fontana MA (2016). Genetic variants associated with subjective well-being, depressive symptoms, and neuroticism identified through genome-wide analyses. Nat Genet.

[49] Smoller JW, Kendler K, Craddock N, Lee PH, Neale BM, Nurnberger JN (2013). Identification of risk loci with shared effects on five major psychiatric disorders: a genome-wide analysis. Lancet.

[50] Hammerschlag AR, Stringer S, De Leeuw CA, Sniekers S, Taskesen E, Watanabe K (2017). Genome-wide association analysis of insomnia complaints identifies risk genes and genetic overlap with psychiatric and metabolic traits. Nat Genet.

[51] Lane JM, Liang J, Vlasac I, Anderson SG, Bechtold DA, Bowden J (2017). Genome-wide association analyses of sleep disturbance traits identify new loci and highlight shared genetics with neuropsychiatric and metabolic traits. Nat Genet.

[52] Furberg H, Kim Y, Dackor J, Boerwinkle E, Franceschini N, Ardissino D (2010). Genome-wide meta-analyses identify multiple loci associated with smoking behavior. Nat Genet.

[53] Rietveld CA, Medland SE, Derringer J, Yang J, Esko T, Martin NW (2013). GWAS of 126,559 individuals identifies genetic variants associated with educational attainment. Science.

[54] Lu Y, Day FR, Gustafsson S, Buchkovich ML, Na J, Bataille V (2016). New loci for body fat percentage reveal link between adiposity and cardiometabolic disease risk. Nat Commun.

[55] Shungin D, Winkler T, Croteau-Chonka DC, Ferreira T, Locke AE, Mägi R (2015). New genetic loci link adipose and insulin biology to body fat distribution. Nature.

[56] Teslovich TM, Musunuru K, Smith AV, Edmondson AC, Stylianou IM, Koseki M (2010). Biological, clinical and population relevance of 95 loci for blood lipids. Nature.

[57] Martin J, Walters RK, Demontis D, Mattheisen M, Lee SH, Robinson E (2018). A genetic investigation of sex bias in the prevalence of attention-deficit/hyperactivity disorder. Biol Psychiatry.

[58] Lagou V, Mägi R, Hottenga JJ, Grallert H, Perry JRB, Bouatia-Naji N (2021). Sex-dimorphic genetic effects and novel loci for fasting glucose and insulin variability. Nat Commun.

[59] Policicchio S, Washer S, Viana J, Iatrou A, Burrage J, Hannon E (2020). Genome-wide DNA methylation meta-analysis in the brains of suicide completers. Transl Psychiatry.

[60] Nagel M, Jansen PR, Stringer S, Watanabe K, De Leeuw CA, Bryois J (2018). Meta-analysis of genome-wide association studies for neuroticism in 449,484 individuals identifies novel genetic loci and pathways. Nat Genet.

[61] Howard DM, Adams MJ, Clarke TK, Hafferty JD, Gibson J, Shirali M (2019). Genome-wide meta-analysis of depression identifies 102 independent variants and highlights the importance of the prefrontal brain regions. Nat Neurosci.

[62] Amare AT, Vaez A, Hsu YH, Direk N, Kamali Z, Howard DM (2020). Bivariate genome-wide association analyses of the broad depression phenotype combined with major depressive disorder, bipolar disorder or schizophrenia reveal eight novel genetic loci for depression. Mol Psychiatry.

[63] Dall’Aglio L, Lewis CM, Pain O (2021). Delineating the genetic component of gene expression in major depression. Biol Psychiatry.

[64] Wu G, Fang YZ, Yang S, Lupton JR, Turner ND (2004). Glutathione metabolism and its implications for health. J Nutr.

[65] Kennedy AJ, Rahn EJ, Paulukaitis BS, Savell KE, Kordasiewicz HB, Wang J (2016). Tcf4 regulates synaptic plasticity, DNA methylation, and memory function. Cell Rep.

[66] Bunger MK, Wilsbacher LD, Moran SM, Clendenin C, Radcliffe LA, Hogenesch JB (2000). Mop3 is an essential component of the master circadian pacemaker in mammals. Cell.

[67] Ge T, Chen CY, Ni Y, Feng YCA, Smoller JW (2019). Polygenic prediction via Bayesian regression and continuous shrinkage priors. Nat Commun.

[68] Goldstein JM, Hale T, Foster SL, Tobet SA, Handa RJ (2019). Sex differences in major depression and comorbidity of cardiometabolic disorders: impact of prenatal stress and immune exposures. Neuropsychopharmacology.

[69] Levey DF, Stein MB, Wendt FR, Pathak GA, Zhou H, Aslan M (2021). Bi-ancestral depression GWAS in the Million Veteran Program and meta-analysis in >1.2 million individuals highlight new therapeutic directions. Nat Neurosci.

[70] Nestler EJ, Carlezon WA (2006). The mesolimbic dopamine reward circuit in depression. Biol Psychiatry.

[71] Pizzagalli DA (2014). Depression, stress, and anhedonia: toward a synthesis and integrated model. Annu Rev Clin Psychol.

[72] Bewernick BH, Kayser S, Sturm V, Schlaepfer TE (2012). Long-term effects of nucleus accumbens deep brain stimulation in treatment-resistant depression: evidence for sustained efficacy. Neuropsychopharmacology.

[73] Krishnan V, Han MH, Graham DL, Berton O, Renthal W, Russo SJ (2007). Molecular adaptations underlying susceptibility and resistance to social defeat in brain reward regions. Cell.

[74] Chaudhury D, Walsh JJ, Friedman AK, Juarez B, Ku SM, Koo JW (2013). Rapid regulation of depression-related behaviours by control of midbrain dopamine neurons. Nature.

[75] Tye KM, Mirzabekov JJ, Warden MR, Ferenczi EA, Tsai HC, Finkelstein J (2013). Dopamine neurons modulate neural encoding and expression of depression-related behaviour. Nature.

[76] Gelenberg AJ, Marlene Freeman CP, Markowitz JC, Rosenbaum JF, Thase ME, Trivedi MH, et al. Practice guideline for the treatment of patients with major depressive disorder. 3rd ed. Work Group on Major Depressive Disorder. 2010.

[77] Ang YS, Kaiser R, Deckersbach T, Almeida J, Phillips ML, Chase HW (2020). Pretreatment reward sensitivity and frontostriatal resting-state functional connectivity are associated with response to bupropion after sertraline nonresponse. Biol Psychiatry.

[78] Barrett ST, Geary TN, Steiner AN, Bevins RA (2017). Sex differences and the role of dopamine receptors in the reward-enhancing effects of nicotine and bupropion. Psychopharmacology (Berl).

[79] Becker JB, Chartoff E (2019). Sex differences in neural mechanisms mediating reward and addiction. Neuropsychopharmacology.

[80] Pruessner JC, Champagne F, Meaney MJ, Dagher A (2004). Dopamine release in response to a psychological stress in humans and its relationship to early life maternal care: a positron emission tomography study using [11C]raclopride. J Neurosci.

[81] Peciña M, Sikora M, Avery ET, Heffernan J, Peciña S, Mickey BJ (2017). Striatal dopamine D2/3 receptor-mediated neurotransmission in major depression: Implications for anhedonia, anxiety and treatment response. Eur Neuropsychopharmacol.

[82] Wang J, Korczykowski M, Rao H, Fan Y, Pluta J, Gur RC (2007). Gender difference in neural response to psychological stress. Soc Cogn Affect Neurosci.

[83] Diekhof EK, Richter A, Brodmann K, Gruber O (2021). Dopamine multilocus genetic profiles predict sex differences in reactivity of the human reward system. Brain Struct Funct.

[84] Gray AL, Hyde TM, Deep-Soboslay A, Kleinman JE, Sodhi MS (2015). Sex differences in glutamate receptor gene expression in major depression and suicide. Mol Psychiatry.

[85] Purgert CA, Izumi Y, Jong YJI, Kumar V, Zorumski CF, O’Malley KL (2014). Intracellular mGluR5 can mediate synaptic plasticity in the hippocampus. J Neurosci.

[86] Shin S, Kwon O, Kang JI, Kwon S, Oh S, Choi J (2015). mGluR5 in the nucleus accumbens is critical for promoting resilience to chronic stress. Nat Neurosci.

[87] Wieroñska JM, Brañski P, Szewczyk B, Paucha A, Papp M, Gruca P (2001). Preliminary communication changes in the expression of metabotropic glutamate receptor 5 (mGluR5) in the rat hippocampus in an animal model of depression. Pol J Pharmacol.

[88] Kovačević T, Skelin I, Minuzzi L, Rosa-Neto P, Diksic M (2012). Reduced metabotropic glutamate receptor 5 in the Flinders Sensitive Line of rats, an animal model of depression: an autoradiographic study. Brain Res Bull.

[89] Śmiałowska M, Szewczyk B, Brański P, Wierońska JM, Pałucha A, Bajkowska M (2002). Effect of chronic imipramine or electroconvulsive shock on the expression of mGluR1a and mGluR5a immunoreactivity in rat brain hippocampus. Neuropharmacology.

[90] Deschwanden A, Karolewicz B, Feyissa AM, Treyer V, Ametamey SM, Johayem A (2011). Reduced metabotropic glutamate receptor 5 density in major depression determined by [(11)C]ABP688 PET and postmortem study. Am J Psychiatry.

[91] Esterlis I, DellaGioia N, Pietrzak RH, Matuskey D, Nabulsi N, Abdallah CG (2018). Ketamine-induced reduction in mGluR5 availability is associated with an antidepressant response: an [11C]ABP688 and PET imaging study in depression. Mol Psychiatry.

[92] Südhof TC (2017). Synaptic neurexin complexes: a molecular code for the logic of neural circuits. Cell.

[93] Hu Z, Xiao X, Zhang Z, Li M (2019). Genetic insights and neurobiological implications from NRXN1 in neuropsychiatric disorders. Mol Psychiatry.

[94] Rucker JJH, Breen G, Pinto D, Pedroso I, Lewis CM, Cohen-Woods S (2013). Genome-wide association analysis of copy number variation in recurrent depressive disorder. Mol Psychiatry.

[95] Tansey KE, Guipponi M, Hu X, Domenici E, Lewis G, Malafosse A (2013). Contribution of common genetic variants to antidepressant response. Biol Psychiatry.

[96] Fabbri C, Tansey KE, Perlis RH, Hauser J, Henigsberg N, Maier W (2018). Effect of cytochrome CYP2C19 metabolizing activity on antidepressant response and side effects: meta-analysis of data from genome-wide association studies. Eur Neuropsychopharmacol.

[97] Trabzuni D, Ramasamy A, Imran S, Walker R, Smith C, Weale ME (2013). Widespread sex differences in gene expression and splicing in the adult human brain. Nat Commun.

[98] Hoffman GE, Ma Y, Montgomery KS, Bendl J, Jaiswal MK, Kozlenkov A (2022). Sex differences in the human brain transcriptome of cases with schizophrenia. Biol Psychiatry.

[99] Hart MP, Hobert O (2018). Neurexin controls plasticity of a mature, sexually dimorphic neuron. Nature.

[100] O’dushlaine C, Rossin L, Lee PH, Duncan L, Parikshak NN, Newhouse S (2015). Psychiatric genome-wide association study analyses implicate neuronal, immune and histone pathways. Nat Neurosci.

[101] Bagot RC, Labonté B, Peña CJ, Nestler EJ (2014). Epigenetic signaling in psychiatric disorders: stress and depression. Dialogues Clin Neurosci.

[102] Peña CJ, Nestler EJ (2018). Progress in epigenetics of depression. Prog Mol Biol Transl Sci.

[103] Penner-Goeke S, Binder EB (2019). Epigenetics and depression. Dialogues Clin Neurosci.

[104] Maze I, Noh KM, Soshnev AA, Allis CD (2014). Every amino acid matters: essential contributions of histone variants to mammalian development and disease. Nat Rev Genet.

[105] Michod D, Bartesaghi S, Khelifi A, Bellodi C, Berliocchi L, Nicotera P (2012). Calcium-dependent dephosphorylation of the histone chaperone DAXX regulates H3.3 loading and transcription upon neuronal activation. Neuron.

[106] Maze I, Wenderski W, Noh KM, Bagot RC, Tzavaras N, Purushothaman I (2015). Critical role of histone turnover in neuronal transcription and plasticity. Neuron.

[107] Hodes GE, Walker DM, Labonté B, Nestler EJ, Russo SJ (2017). Understanding the epigenetic basis of sex differences in depression. J Neurosci Res.

[108] Miller AH, Raison CL (2016). The role of inflammation in depression: from evolutionary imperative to modern treatment target. Nat Rev Immunol.

[109] Musselman DL, Evans DL, Nemeroff CB (1998). The relationship of depression to cardiovascular disease: epidemiology, biology, and treatment. Arch Gen Psychiatry.

[110] Anderson RJ, Freedland KE, Clouse RE, Lustman PJ (2001). The prevalence of comorbid depression in adults with diabetes: a meta-analysis. Diabetes Care.

[111] Naqvi TZ, Naqvi SSA, Merz CNB (2005). Gender differences in the link between depression and cardiovascular disease. Psychosom Med.

[112] Heo M, Pietrobelli A, Fontaine KR, Sirey JA, Faith MS (2006). Depressive mood and obesity in US adults: comparison and moderation by sex, age, and race. Int J Obes.

[113] Möller-Leimkühler AM (2007). Gender differences in cardiovascular disease and comorbid depression. Dialogues Clin Neurosci.

[114] Goldstein JM, Holsen L, Huang G, Hammond BD, James-Todd T, Cherkerzian S (2016). Prenatal stress-immune programming of sex differences in comorbidity of depression and obesity/metabolic syndrome. Dialogues Clin Neurosci.

[115] Marcus SM, Kerber KB, Rush AJ, Wisniewski SR, Nierenberg A, Balasubramani GK (2008). Sex differences in depression symptoms in treatment-seeking adults: confirmatory analyses from the Sequenced Treatment Alternatives to Relieve Depression study. Compr Psychiatry.

[116] Wang GJ, Volkow ND, Logan J, Pappas NR, Wong CT, Zhu W (2001). Brain dopamine and obesity. Lancet.

[117] Hyde CL, Nagle MW, Tian C, Chen X, Paciga SA, Wendland JR (2016). Identification of 15 genetic loci associated with risk of major depression in individuals of European descent. Nat Genet.

