# Supplementary material for: A sex-specific genome-wide association study of depression phenotypes in UK Biobank
Source: Mol Psychiatry. 2023 Feb 7;28(6):2469–79. doi: 10.1038/s41380-023-01960-0 (PMC10611579; doi:10.1038/s41380-023-01960-0)
Supplement: Supplementary file 1 — Supplementary tables [file 41380_2023_1960_MOESM1_ESM.doc]

**Supplementary Tables**

**A Sex-Specific Genome-Wide Association Study of Depression Phenotypes in UK Biobank**

Patrícia Pelufo Silveira, MD, PhD, Irina Pokhvisneva, MSc, David M Howard, PhD, Michael J. Meaney, PhD

**Table S1 -** The study demographics for the case and control groups within the broad MDD UK Biobank phenotype. Data is expressed as means and standard deviations or percentages and number of participants.

**A**

| **Total** | **Total** | **Control** | **MDD broad** |
| --- | --- | --- | --- |
| Number of subjects | 274141 | 177152 | 96989 |
| Sex |  |  |  |
| Female | 53.40% | 47.50% | 64% |
| Male | 46.60% | 52.50% | 36% |
| Mean age at recruitment | 56.8 (7.98) | 57.1 (8.06) | 56.4 (7.80) |
| Townsend deprivation index | -1.61 (2.92) | -1.77 (2.82) | -1.31 (3.07) |
| MDD broad % | 35.40% |  |  |

**B**

| **Males** | **Total** | **Control** | **MDD broad** |
| --- | --- | --- | --- |
| Number of subjects | 127867 | 92944 | 34923 |
| Mean age at recruitment | 57.1 (8.08) | 57.2 (8.15) | 56.7 (7.9) |
| Townsend deprivation index | -1.57 (2.98) | -1.72 (2.88) | -1.17 ( 3.2) |
| MDD broad % | 27.30% |  |  |

**C**

| **Females** | **Total** | **Control** | **MDD broad** |
| --- | --- | --- | --- |
| Number of subjects | 146274 | 84208 | 62066 |
| Mean age at recruitment | 56.6 (7.88) | 56.9 (7.96) | 56.2 (7.74) |
| Townsend deprivation index | -1.64(2.87) | -1.82 (2.76) | -1.39 (2.99) |
| MDD broad % | 42.40% |  |  |

**Table S2 -** Estimates of the intercept, standard error and genomic inflation factor obtained from linkage disequilibrium score regression

| Broad MDD GWAS | Intercept | Standard Error | Genomic inflation |
| --- | --- | --- | --- |
| **Total** | 1.0085 | 0.0076 | 1.2932 |
| **Females** | 1.0104 | 0.0066 | 1.1811 |
| **Males** | 1.0025 | 0.0066 | 1.127 |

**Table S3 -** Baseline characteristics for test sample. Data is expressed as means and standard deviations or percentages and number of participants.

|  | **Total** | **Control** | **MDD broad** |
| --- | --- | --- | --- |
| Number of subjects | 65285 | 42038 | 23247 |
| Sex |  |  |  |
| Female | 55.40% | 49.70% | 65.80% |
| Male | 44.60% | 50.30% | 34.20% |
| Mean age at recruitment | 57.1 (7.97) | 57.4 (8.03) | 56.6 (7.82) |
| Townsend deprivation index | -1.48 (2.95) | -1.65 (2.86) | -1.16 (3.09) |
| MDD broad % | 35.60% |  |  |

**Table S4 –** Estimated effect between sex-specific broad MDD GWAS PRS and log CRP levels

| **Outcome** | **Predictor** | **P value** | **Beta** | **SE Beta** |
| --- | --- | --- | --- | --- |
| *Females, N=34,789* | | | | |
| logCRP levels | Female PRS pT 0.0001 | 0.0005 | 0.0198 | 0.0057 |
|  | Female PRS pT 0.001 | 0.0001 | 0.0218 | 0.0057 |
|  | Female PRS pT 0.01 | 0.0006 | 0.0198 | 0.0058 |
|  | Female PRS pT 0.05 | 0.0001 | 0.0220 | 0.0058 |
|  | Female PRS pT 0.1 | 0.0004 | 0.0205 | 0.0058 |
|  | Female PRS pT 0.2 | 0.0006 | 0.0200 | 0.0058 |
|  | Female PRS pT 0.5 | 0.0008 | 0.0196 | 0.0058 |
| *Males, N=28,015* | | | | |
| logCRP levels | Male PRS pT 0.0001 | 0.2550 | 0.0069 | 0.0060 |
|  | Male PRS pT 0.001 | 0.5452 | 0.0036 | 0.0060 |
|  | Male PRS pT 0.01 | 0.3866 | 0.0052 | 0.0060 |
|  | Male PRS pT 0.05 | 0.7586 | -0.0019 | 0.0061 |
|  | Male PRS pT 0.1 | 0.6463 | 0.0028 | 0.0061 |
|  | Male PRS pT 0.2 | 0.6122 | 0.0031 | 0.0061 |
|  | Male PRS pT 0.5 | 0.7172 | 0.0022 | 0.0061 |
